# Technical Brief: A novel strategy for enrichment of trabecular meshwork protease proteome

**Published:** 2008-05-14

**Authors:** Renata Picciani, Anna K. Junk, Sanjoy K. Bhattacharya

**Affiliations:** Bascom Palmer Eye Institute, University of Miami, Miami, FL

## Abstract

We present a novel and simple enrichment strategy to capture trabecular meshwork (TM) protease proteome. The method relies on fractionation of TM tissue into cytosolic and nuclear extracts and subsequent affinity enrichment of proteases on peptide inhibitors. A large repertoire of available protease substrate analog peptides enables an improved capture of TM protease proteome compared to SDS–PAGE fractionation alone. Peptide analog inhibitors of protease substrates are immobilized on a protein A or G column using 254 nm intense ultraviolet (UV) light. The TM cytosolic protein extract incubated on the column is eluted with salt or a buffer with a low hydrogen ion concentration. The resultant protein solution is precipitated with acetone, fractionated on SDS–PAGE, in situ trypsin digested, and subjected to mass spectrometry. This relatively simple protocol enables improved capture of cytosolic proteases. We identified 20 previously reported TM proteins from a single donor tissue using affinity enrichment. The majority of identified proteins were either intracellular proteases or known protease inhibitors. Both serine and cysteine proteases were captured using this strategy with improved coverage compared to our previous identification without affinity enrichment.

## Introduction

The glaucomas are a group of irreversible blinding optic neuropathies that affect over 70 million people worldwide [1]. Glaucoma is often associated with increased intraocular pressure (IOP) leading to damage of the optic nerve. The imbalance of aqueous humor production and outflow results from IOP elevation. The aqueous humor is actively produced by the ciliary body epithelium and exits the eye through the structures of the anterior chamber angle after bathing the lens and the cornea. The aqueous humor outflow experiences most resistance at the level of trabecular meshwork (TM) [2]. In open-angle glaucoma, poorly understood structural and functional changes in the TM such as remodeling of the TM extracellular matrix (ECM) are associated with increased resistance, which impedes the aqueous outflow [3-5]. The ECM changes in the TM have been suggested by several biochemical studies [4,6,7] and directly supported by ultrastructural studies [5,8]. Changes and/or remodeling of the ECM can occur in a variety of ways such as increased secretion and decreased degradation of matrix proteins, cell adhesion, migration, and changes in cell shape and number [7,9]. Degradation of ECM components by matrix metalloproteases enables changes in cell morphology, adhesion, and migration. Increased secretion and/or secretion of altered forms of proteins including matrix metalloproteases are approaches employed by cells in ECM remodeling as well. The precise role of matrix metalloproteases in the ECM remodeling has been the subject of several studies [10-12]. However, intracellular proteases also have the potential to provoke ECM changes by processing expressed matrix gene products [13,14] and modifying different forms of receptors/transporters that are important for entry and exit of key cellular homeostatic components such as the endothelin B receptor [15]. However, comprehensive studies in changes of TM intracellular proteases have not been performed. Recent investigations suggest that important alterations including impairment of intracellular proteases due to posttranslational modifications occur in the glaucomatous TM [6,14]. The changes in the intracellular protease proteome can be identified using high-throughput proteomic mass spectrometric approaches. One of the challenges in proteomic studies lies in reducing the complexity in capturing, identifying, and eventually quantifying the proteome changes [16]. Here, we present a simple and efficient method that allows better capturing of TM intracellular proteases. This method will enhance our understanding of TM intracellular protease changes that occur as part of glaucoma pathophysiology.

## Methods

### Tissue procurement

Normal eyes (Table 1) were procured from the National Disease Research Institute (Philadelphia, PA) and the Lions Eye Bank (Miami, FL). The eyes were enucleated within 10 h of death, placed in a moisture chamber at 4 °C, and transported. These eyes were dissected within 48 h, and the TM was carefully excised for study. Fresh porcine TM tissue was isolated from freshly enucleated eyes from euthanized pigs procured from the University of Miami Department of Surgery following IACUC approved protocols.

**Table 1:** Donor details.

**Age**	**Race**	**Gender**	**Time of death to enucleation (h)**
55	W	M	8
53	W	M	7
62	W	F	10
76	W	F	9
55	W	M	9

The Caucasian race is indicated by W. Letters M and F denote male and female, respectively.

### Preparation of cytosolic extract

TM was carefully dissected from normal cadaver eyes. TM cytosolic and nuclear protein extracts were obtained using the NE-PER Nuclear and Cytoplasmic Extraction Reagents kit (Cat number 78833; Pierce Biotechnology, Rockford, IL) and following the protocols recommended by the manufacturer. The recovered proteins were subjected to spectrophotometric quantification using the Bradford assay and subsequently aliquoted for use or stored at −80 °C for future analysis. All protein aliquots were either freshly used or subjected to only one freeze–thaw cycle.

### Western blot analyses

Approximately 10 µg of total cytosolic and nuclear protein extracts were fractionated on a 4%–20% Tris-glycine gradient gel (Invitrogen Corporation, Carlsbad, CA), transferred onto a polyvinylidene fluoride (PVDF) membrane, and incubated overnight at 4 °C with the antibodies (~5 μg/ml) detailed below. For these analyses, rabbit monoclonal antibody against histone H3 (Cat number 05–928; Upstate^®^, Billerica, MA) and rabbit polyclonal antibody against GAPDH (Cat number sc-25778; Santa Cruz Biotechnology Inc., Santa Cruz, CA) were used. Subsequently, a 2 h incubation at 4 °C with the appropriate horseradish peroxidase secondary antibodies was performed. All incubations occurred in 5% milk. Detection was performed using electrochemiluminescence (ECL; catalog number 32106; Pierce Biotechnology, Rockford, IL).

### Preparation of enrichment column and capture of proteases

The enrichment column was prepared by ultraviolet (UV) cross-linking of peptides present in commercial protease inhibitor cocktails. Two different protease inhibitors (100 µl each; Protease inhibitor cocktail for use with mammalian cell and tissue extracts and Sigma Fast Protease inhibitor tablets for general use, Cat number P8340 and S8820, respectively; Sigma-Aldrich Inc., St. Louis, MO) were incubated with 200 µl (2.5 mg) of Protein A Sepharose beads (Cat number 170780–01; Amersham Biosciences, Piscataway, NJ) for 15 min and subsequently UV cross-linked for 90 s at 120 mJ/cm^2^ in a UV transilluminator equipped with 254 nm bulbs. A cytosolic protein extract was added (~250 µg) and incubated for 1 h at 4 °C with gentle shaking. For the controls, no protease inhibitor was added to the sample (two different protease inhibitor cocktails, which slightly differ in their composition of inhibitory peptides, were used to rule out the possibility that the capture of proteases is not vastly different between the two). A 2 ml column (Cat number 89896; Pierce Biotechnology, Rockford, IL) was equilibrated with 1 ml 1X PBS. The samples were passed through the column three times and then washed with 250 µl 1X PBS twice. Subsequently, 100 µl of 1 M NaCl plus 100 mM KCl were used for elution. Finally, the elution products were subjected to acetone precipitation with the addition of four volumes of cold acetone and to incubation at room temperature for 15 min with or without carrier tRNA (Yeast tRNA; Catalog number 15401029; Invitrogen Inc., Carlsbad, CA). Carrier tRNA was added to enhance the capture of proteins during acetone precipitation. The samples were then centrifuged at 12,000 rpm for 20 min. The supernatant was discarded, and the pellet was resuspended in 5 µl of water.

### Gel electrophoretic fractionation and mass spectrometry

The fractionation of elution products was performed either on a 4%–20% Tris-glycine gradient gel (Invitrogen Corporation, Carlsbad, CA) or on a 4%–15% gradient PHAST gel (Cat number 17–0678–01; GE Healthcare Biosciences Corp, Piscataway, NJ). The gel was subjected to Coomassie Blue staining followed by SyproRuby Protein Gel Stain (Cat number S12001; Invitrogen Corporation, Carlsbad, CA) and silver staining (Pro-Pure protein assay, Cat number M2271Lkit; AMRESCO Inc., Solon, Ohio). Approximately, 10 µg of the TM protein extracts (total cytosolic and nuclear extract) was also loaded on the 4%–20% Tris-glycine gel. On 4%–15% PHAST gel, 4 µg of cytosolic extract was also loaded in addition to eluents.

For mass spectrometric identification of proteins, gel slices were excised and digested in situ with sequencing grade porcine trypsin (Promega Biosciences Inc., San Luis Obispo, CA). The products of in situ digestion were loaded onto pre-columns 360 mm od x 100 mm id fused silica (Polymicro Technologies, Phoenix, AZ) packed with 3 cm of irregular 5–15 µm-non-spherical C18 (YMC Inc., Wilmington, NC). The column was washed with 0.1 M acetic acid (AcOH) for 5 min before switching in-line with the resolving column (7-cm spherical C18, 360 od x 100 id). Once the columns were in-line, the peptides were gradient eluted with a 0%–100% B in 30 min where A was 0.1 M AcOH in nanopure water and B was 0.1 M AcOH in 80% acetonitrile. All samples were analyzed using a Thermo Electron Finnigan LTQ (San Jose, CA). Electrospray was accomplished using an Advion Triversa Nanomate (Advion Biosystems, Ithaca, NY) with a voltage of 1.7 kV and a flow rate of 250 nl/min. The mass spectrometer was operated in data dependent mode with the top five most abundant ions in each spectrum being selected for sequential tandem mass spectrometry (MS/MS) experiments. The exclusion list was used (1 repeat, 180 s return time) to increase the dynamic range. All MS/MS spectra were searched with Sequest version 2.7 (ThermoFinnigan, San Jose, CA) and Mascot using NCBI non-redundant, Ensemble, and SwissProt databases. The search of database entries were restricted to Human and Metazoa (animals) and allowed for a maximum of two missed cleavages. The protonated molecule ions “MH+” and “Monoisotopic” were defined for the peak mass data input. The SEQUEST Sp and Xcorr cutoff scores of the protonated “MH+” peptides were 500 and 1.8, respectively. For Mascot searches, the score was greater than 78. All spectra were visually inspected for determination of correct database assignment. The potential chemical modifications of a peptide such as alkylation of a cysteine (carbamidomethyl [C]), oxidation of a methionine residue (M), and acetylation of a lysine (K) were also considered in the search. No restrictions were imposed for either the protein isoelectric point or the molecular weight during the search.

## Results

### Enrichment of proteins in immobilized protease inhibitor peptide columns

**Figure 1:**
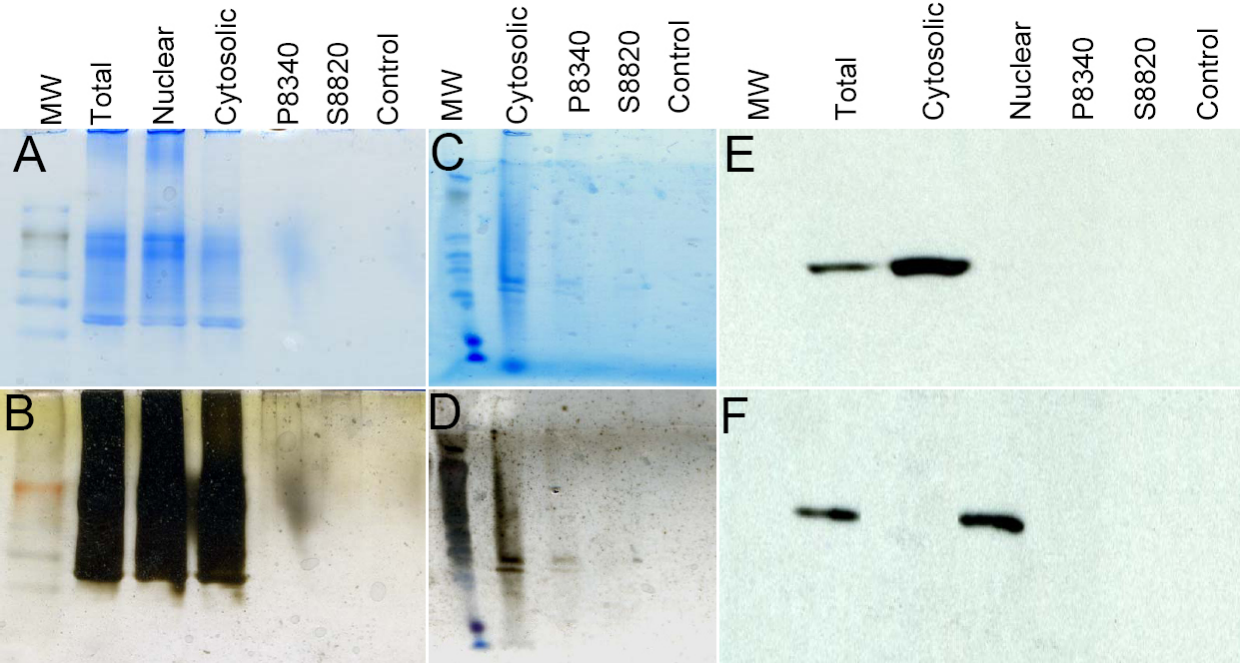
Analyses of SDS–PAGE fractionated proteins in enrichment steps. **A.** About 100 µg of protein (initial load) was subjected to affinity enrichment on P8340 or S8820 column as indicated and total eluted and acetone precipitated proteins were subjected to SDS–PAGE fractionation and stained with Coomassie blue. An initial load (10 µg) of total, nuclear and cytosolic proteins were fractionated on a SDS–PAGE. For this purpose cytosolic and nuclear fractions (10 µg proteins each) from NE-PER Nuclear and Cytoplasmic Extraction Reagents kit (Pierce Biotechnology Inc.) were obtained **B.** The same gel as in A was subjected to silver staining. **C.** About 250 µg of protein (initial load) was subjected to affinity enrichment on P8340 or S8820 column as indicated. Total eluted and acetone precipitated proteins were subjected to fractionation on a 4%–15% PHAST gel (GE Healthcare) and stained with Coomassie blue. **D.** The same gel as in C was subjected to silver staining. The control is the cytosolic fraction (4 µg) passed through an empty protein A column as described in methods. **E.** After transfer to a PVDF membrane, western analyses of protein extracts were performed, using GAPDH antibody and, **F.** Histone H3 antibody, as described in methods.

Human TM samples were solubilized using buffers provided in the NE-PER Nuclear and Cytoplasmic Extraction Reagents kit (Pierce Biotechnology Inc.) and subjected to fractionation into cytosolic and nuclear fractions as described in Methods. The cytosolic fraction was subjected to affinity enrichment for proteases over a P8340 or a S8820 peptide inhibitor cocktail column. The column affinity enrichment resulted in the recovery of very small amounts of proteins. SDS–PAGE fractionation of proteins (4%–20% gradient mini gels; Invitrogen Inc.) did not allow detection of protein bands upon Coomassie Blue (Figure 1A) or silver staining (Figure 1B). The same amount of eluted proteins in a final volume of 4 µl was loaded onto a six-well PHAST gel (GE Healthcare) and subjected to fractionation and staining. Both Coomassie blue and silver staining allowed detection of the eluted bands (Figure 1C,D). To ensure the fractionation of cytosolic and nuclear proteins, the total protein, cytosolic, and nuclear fractions were subjected to western blot analyses (Figure 1E,F). When the blot was probed with antibody against GAPDH, only total and cytosolic extracts showed presence of GAPDH immunoreactivity (Figure 1E). On the other hand, probing the blot with histone H3 antibody revealed reactivity in the total and nuclear extracts and not in the cytosolic extract (Figure 1F), suggesting fractionation of total proteins into cytosolic and nuclear extracts.

### Quantification of peptide column recovered proteins

**Figure 2:**
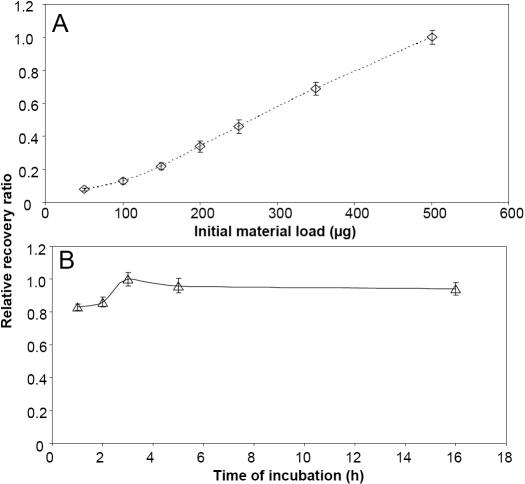
Effect of Initial material and time of incubation on relative recovery of proteins from immobilized affinity peptide columns. **A.** The relative recovery of protein was determined using fractionated Porcine cytosolic proteins on a PHAST gel using silver staining and densitometric scan of the area. The densitometric scan of product from 550 µg of initial TM extract incubated with beads for 3 h, recovered using 1 M NaCl, 100 mM KCl elution after acetone precipitation using 10 µg of carrier yeast tRNA as reference (considered as 100 percent) was used for determination of relative recovery ratios. **B.** Porcine TM extracts (500 µg initial load) was used for incubation at different times as indicated was eluted with 1 M NaCl and 100 mM KCl using carrier yeast tRNA. Results of three independent determinations were used to determine the mean and standard deviation shown here.

The method presented here is to enable the capture of the protease proteome profile from single tissue samples. This method is aimed to eventually be applied to surgical TM tissue samples. While it is possible to quantify the proteins recovered from immobilized peptide columns with established biochemical methods for large initial protein loads (milligram quantities), the limited initial protein load in actual tissue samples prohibits quantification with the current biochemical methods (i.e., Bradford, Folin-Ciocalteau, Lowry, and Amido Black). For this purpose, we have performed experiments with porcine TM tissue to reduce or limit the use of precious human tissue for method development alone. Our studies indicate that reproducible relative quantification is possible for fractionated proteins on the PHAST gel system using silver staining and densitometry, but absolute quantification is not. Using porcine TM extract and densitometric scans of silver staining, the impact of the initial material (within a range of 50–500 µg) and time of incubation (1–16 h) was found to have an effect on the relative recovery of proteins (Figure 2). The densitometric scan of the recovered products from 550 µg of initial TM extract was incubated with immobilized peptide beads for 3 h, eluted with 1 M NaCl plus 100 mM KCl followed by acetone precipitation; 10 µg of carrier yeast tRNA was used as a reference (considered as 100 percent). This reference was used for calculation of the relative recovery ratios for all other elutions. To determine the effect of incubation time on relative recovery of eluted products, an initial load of 500 µg of porcine TM and was used, and elution was performed in an identical fashion as noted above.

### Enhanced recovery as a function of carrier molecules and/or electrolyte addition

The recovery of proteins within a 50–250 µg range of initial material was found to be enhanced with the use of carrier molecules (yeast t-RNA). The electrolytes, a combination of sodium and potassium, have an effect on the recovery of silver stained proteins. The addition of potassium chloride increased the recovery of proteins (data not shown). The addition of potassium chloride and carrier tRNA also reduced batch to batch variation in recovery especially with low initial loads (50–250 µg).

### Mass spectrometric identification of recovered trabecular meshwork proteases

In normal TM, a total of 20 proteins were identified (Table 2). The majority of the identified proteins are proteases encompassing both serine and cysteine proteases (Table 2). Several protease inhibitors such as anti-chymotrypsin, anti-trypsin, and anti-thrombin were also identified (Table 2). Some non-protease proteins were also identified (Table 2). At this point, it is not well understood why these proteins/peptides showed strong binding. Proteases can bind to peptides, which are not their known high affinity substrate analog. Consequently, the TM proteases identified by affinity enrichment may or may not have a direct peptide inhibitor/analog. We identified all proteases from one single TM tissue in our current analyses that were identified from combined multiple numbers of TMs by proteomic analysis in a previous study [17]. The enhancement of identification in the current study is due to affinity enrichment of proteases. Although the total number of intracellular proteases identified are the same in our previous and in our current analyses (Table 2), they are different in two respects. First, all identified proteases are from a single TM tissue sample rather than from multiple TM samples, and second, all identified protease peptide matches (coverage) are greater than our previous analysis. In the previous analyses, fewer proteases were identified from a single TM tissue. All the proteases enlisted in Table 2 were identified only when six different TM samples were combined [17]. There are some non-protease proteins (for example, protease inhibitor anti-trypsin) for which fewer peptides were identified in the current analysis than in our previous analysis. However, overall a better capture of protease peptides was obtained with affinity enrichment as described above.

**Table 2:** Select proteases and proteins identified from normal trabecular meshwork.

**Protein**	**Accession number ^1^**	**Peptide matches^2^**	**Peptide matches^3^**	**Donors^3^**
Alpha-1-antichymotrypsin precursor ^4^	P01011	4	5	6
Alpha-1-antitrypsin precursor^4^	P01009	4	14	6
Antithrombin-III precursor^4^	P01008	3	2	3
Calpactin I light chain^5^	P08206	7	1	1
Calpain 1, large [catalytic] subunit	P07384	7	6	3
Calponin H1, smooth muscle^5^	P51911	3	1	2
Caspase-14 precursor	P31944	6	2	3
Cathepsin D^6^	P07339	6	2	2
Ceruloplasmin precursor^5^	P00450	5	5	4
Coagulation factor XIII A chain^5^	P00488	2	1	1
Complement C3^7^	P01024	4	3	3
Complement C4 precursor^7^	P01028	4	1	2
Complement component 1^5,7^	Q07021	3	2	1
Complement factor B precursor^7^	P00751	3	2	1
Complement factor H precursor^7^	P08603	3	2	1
Endoplasmin^5^	P14625	3	2	3
Neurolysin, mitochondrial	Q9BYT8	2	1	2
Prothrombin precursor	P00734	2	1	3
Puromycin-sensitive aminopeptidase	P55786	3	1	1
Tripeptidyl-peptidase II	P29144	3	1	1

The proteins that mostly proteases identified by LC MS/MS of protein extracts were derived from the TM of a 55-year-old male Caucasian donor after enrichment on an immobilized immunocolumn and SDS–PAGE fractionation as described in Methods. ^1Swiss-Prot^ database entries are shown; ^2^peptide matches in current mass spectrometric analyses; ^3^peptide matches and number of donors from our previous TM proteomic analyses [17] without affinity enrichment; ^4^identified protease inhibitors; ^5^identified proteins that are not proteases; ^6^cathepsin D has been identified as bovine and human protein in our previous and current TM proteomic analysis, here human protein accession number has been provided; ^7^not all complement components possess proteolytic activity; however, they act as a complex which results in proteolytic activity, here we have placed them in the protease category.

## Discussion

Glaucoma is thought to be due to increased resistance at the TM, which impedes aqueous humor outflow. Changes in the ECM are responsible for the altered resistance. Increased production of ECM proteins or, alternatively, processed secreted protein products may result in changes in the matrix composition and in ECM remodeling. Several factors such as elevated pressure or mechanical stretching have also been shown to influence ECM gene expression of the TM in vivo and in cultured TM cells [13,18]. While the role of matrix metalloproteases has been well studied in TM remodeling [4,10], changes in the TM intracellular proteases have been the subject of scant studies. Cellular proteasome have been found to undergo oxidative modification, resulting in the impairment of their function in the glaucomatous TM [6,19,20]. A comprehensive and quantitative investigation of the TM proteasomal activities also remains to be performed.

The proteomic analysis of clinically relevant tissues requires access to fresh or frozen specimens with adequate morphological consistency and sufficient quantity as well as proper and detailed clinical records [21]. The availability of such properly preserved tissues is often scarce especially ocular tissues. This fact limits meaningful outcome of proteomic analyses. Obtaining sufficient quantity of different ocular tissues is important for proteomic and genomic analyses, one providing complementary information to the other. Enrichment of proteins is a necessity due to the tiny nature of tissue regions in the case of ocular tissues. The TM is one such region where the availability of the amount of tissue material from a single donor is often limited (due to the tiny nature of the region), and without enrichment, successful identification of protease proteome will be severely limited.

Identification of proteins by mass spectrometry can be limited by several factors even for very sensitive mass spectrometers. Some of these factors are: a masking effect by other, abundant proteins with similar isoelectric points, overlay by proteins that undergo better digestion and thus generating peptides in the similar isoelectric point range, and finally, peptides more susceptible to ionization and thus coming in close proximity to mass/charge ratio with respect to peptides of interest. The reduction of the complexity of the protein mixture (in protein extracts) is an important aspect in the mass spectrometric identification of proteins. Immobilized inhibitor or substrate analog entrapment of enzymes is an important means for enrichment of enzymes including proteases which reduce complexity while enriching the proteins. Several other fractionation techniques have been used in an attempt to reduce the complexity in conjunction with affinity enrichment, including 2D blue native gel electrophoresis which resolves protein complexes in a non-denaturing first dimension gel followed by the fractionation of the complex components in a denaturing second dimension gel [22].

The immobilized inhibitors or inhibitory peptides have been in use for affinity purification. Immobilized peptide arrays have been used for epitope mapping as well as protein–protein interaction, enzyme-substrate interaction, and enzyme-inhibitor interaction studies [23]. Inhibitor affinity chromatography, using immobilized substrate analogs or other inhibitors, has been employed to isolate enzyme proteomes and is often referred to as the chemiproteomic approach [24]. Affinity enrichment of many different enzymes such as kinases, phosphatases, and matrix metalloproteases (MMPs) have benefited from the use of this approach [24-28]. However, such approaches are yet to be applied to the comprehensive enrichment of intracellular proteases, particularly in the ocular tissues [28,29]. Immobilized inhibitors have not only been used for affinity enrichment but also for several other novel uses [30-32]. Surface-immobilized, oriented peptide aptamers have been used for the detection of specific target proteins in complex biologic solutions [27]. Aprotinin-linked fibrinogen in a novel approach has been used for controlling degradation of fibrin gels [32]. The precursor state of factor Xa heparin-binding exosite was revealed by Ixolaris binding to factor X in a novel fashion [31]. Label-free, high-throughput functional lytic assays based on immobilized inhibitors or substrates have made the development of refractive index-sensitive resonant waveguide grating biosensors possible [30]. Affinity enrichment of MMPs is a routine procedure for non-ocular tissues [28,29]. The approach outlined here can be used in combination with immobilized MMP target peptides and immobilized metal and gelatin GQ columns to enable the capture of MMPs and their tissue inhibitors. Sequential capture of matrix and intracellular proteases from the same tissue is possible by combination of these techniques, maximizing the utility of tissue toward comprehensively discovering changes in the protease proteome composition in glaucoma (or any other disease) in comparison to the normal state. Additives or carrier molecules, which are usually proteins, electrolytes, and tRNAs, enhance recovery of biomacromolecules including proteins and DNA [33,34]. The carrier molecules usually act as chaperones and help entrap low amounts of protein following elution. Electrolytes typically promote charge-charge interaction and help in coagulation thus facilitating enhanced recovery. We have used two different inhibitor peptide cocktails to determine whether there are vast differences in their efficiency to capture target proteins. tRNA was added to enhance the recovery of proteins while it serves as a carrier molecule during acetone precipitation.

Compared to our previous analyses [17], current analyses captured a greater number of protease peptides from a single TM sample, demonstrating the utility and efficacy of the fractionation strategy presented here. Several serine and cysteine proteases have thus been identified (Table 2). In particular, several complement components have also been identified. It is important to note that complement components have been found to impair synapses and have been implicated in neurodegeneration of retinal ganglion cells, a process that is thought to occur in glaucoma [35]. Our analyses also revealed the release and capture of mitochondrial neurolysin and tripeptidyl peptidase II (Table 2). Tripeptidyl peptidase II is a component of the proteolytic cascade acting downstream of the 26S proteasome in the ubiquitin-proteasome pathway [36,37]. Tripeptidyl peptidase II is thought to be able to complement the 26S proteasome function under conditions in which the latter is inhibited. Therefore, the expression of tripeptidyl peptidase II in normal TM suggests that perhaps in at least some eyes, the proteasomal function is impaired long before the pathology is apparent. Our efforts to capture protease proteome in the DBA/2J mouse model using combinatorial affinity enrichment methods outlined above is under progress to further our understanding of these proteases and their alterations.
